# Pre-clinical studies in cough research: Role of Transient Receptor Potential (TRP) channels

**DOI:** 10.1016/j.pupt.2013.02.007

**Published:** 2013-10

**Authors:** Megan S. Grace, Eric Dubuis, Mark A. Birrell, Maria G. Belvisi

**Affiliations:** a Respiratory Pharmacology, Pharmacology & Toxicology Section, National Heart and Lung Institute, Faculty of Medicine, Imperial College London, Exhibition Road, London SW7 2AZ, UK; b Respiratory Research Group, University of Manchester, Wythenshawe Hospital, Southmoor Road, Manchester M23 9LT, UK

**Keywords:** Cough, Sensory nerve, Transient receptor potential, Respiratory pharmacology, Airway disease

## Abstract

Cough is a protective reflex and defence mechanism in healthy individuals, which helps clear excessive secretions and foreign material from the lungs. Cough often presents as the first and most persistent symptom of many respiratory diseases and some non-respiratory disorders, but can also be idiopathic, and is a common respiratory complaint for which medical attention is sought. Chronic cough of various aetiologies is a regular presentation to specialist respiratory clinics, and is reported as a troublesome symptom by a significant proportion of the population. Despite this, the treatment options for cough are limited. The lack of effective anti-tussives likely stems from our incomplete understanding of how the tussive reflex is mediated. However, research over the last decade has begun to shed some light on the mechanisms which provoke cough, and may ultimately provide us with better anti-tussive therapies. This review will focus on the *in vitro* and *in vivo* models that are currently used to further our understanding of the sensory innervation of the respiratory tract, and how these nerves are involved in controlling the cough response. Central to this are the Transient Receptor Potential (TRP) ion channels, a family of polymodal receptors that can be activated by such diverse stimuli as chemicals, temperature, osmotic stress, and mechanical perturbation. These ion channels are thought to be molecular pain integrators and targets for novel analgesic agents for the treatment of various pain disorders but some are also being developed as anti-tussives.

## 1 Introduction

The cough reflex forms part of the body's immune defence against inhaled substances and invading pathogens. Under normal conditions, coughing serves to stop potentially harmful substances from being inhaled as well as clearing excessive secretions and foreign material from the airways [1]. A persistent cough is therefore often the first sign of respiratory diseases such as the common cold, lung infections, asthma, chronic obstructive pulmonary disease (COPD), pulmonary fibrosis and lung cancer. Moreover, cough can be associated with non-respiratory disorders such as gastro-oesophageal reflux and postnasal drip; and can also be idiopathic, where the cough is not associated with any discernible underlying disorder [2–4]. Under pathological conditions, the cough reflex can be sensitised to both noxious and innocuous stimuli, and is often non-productive. This leads to excessive or chronic coughing, which can become painful due to mechanical damage to the upper airways [5]. Other detrimental effects commonly associated with chronic cough also severely affect quality of life, such as anxiety, incontinence, sleep disturbance and depression [6–8].

Unfortunately, current over-the-counter anti-tussive medicines are not thought to be efficacious [9,10]; and the current gold standard in cough therapy (opiates or their derivatives) are associated with numerous side effects [11–15]. Furthermore, cough treatments are potentially dangerous for young children and are not recommended for use by children under 2 years of age [15,16]. The ideal anti-tussive therapy would inhibit an enhanced and problematic cough without affecting the normal protective reflex that is associated with health benefits. Novel cough therapies that are both safe and efficacious are therefore urgently required. Scientists have recently begun to elucidate the mechanisms that drive the cough reflex, which will hopefully lead to better anti-tussive treatments in the near future.

## 2 Airway sensory nerves

**Fig. 1:**
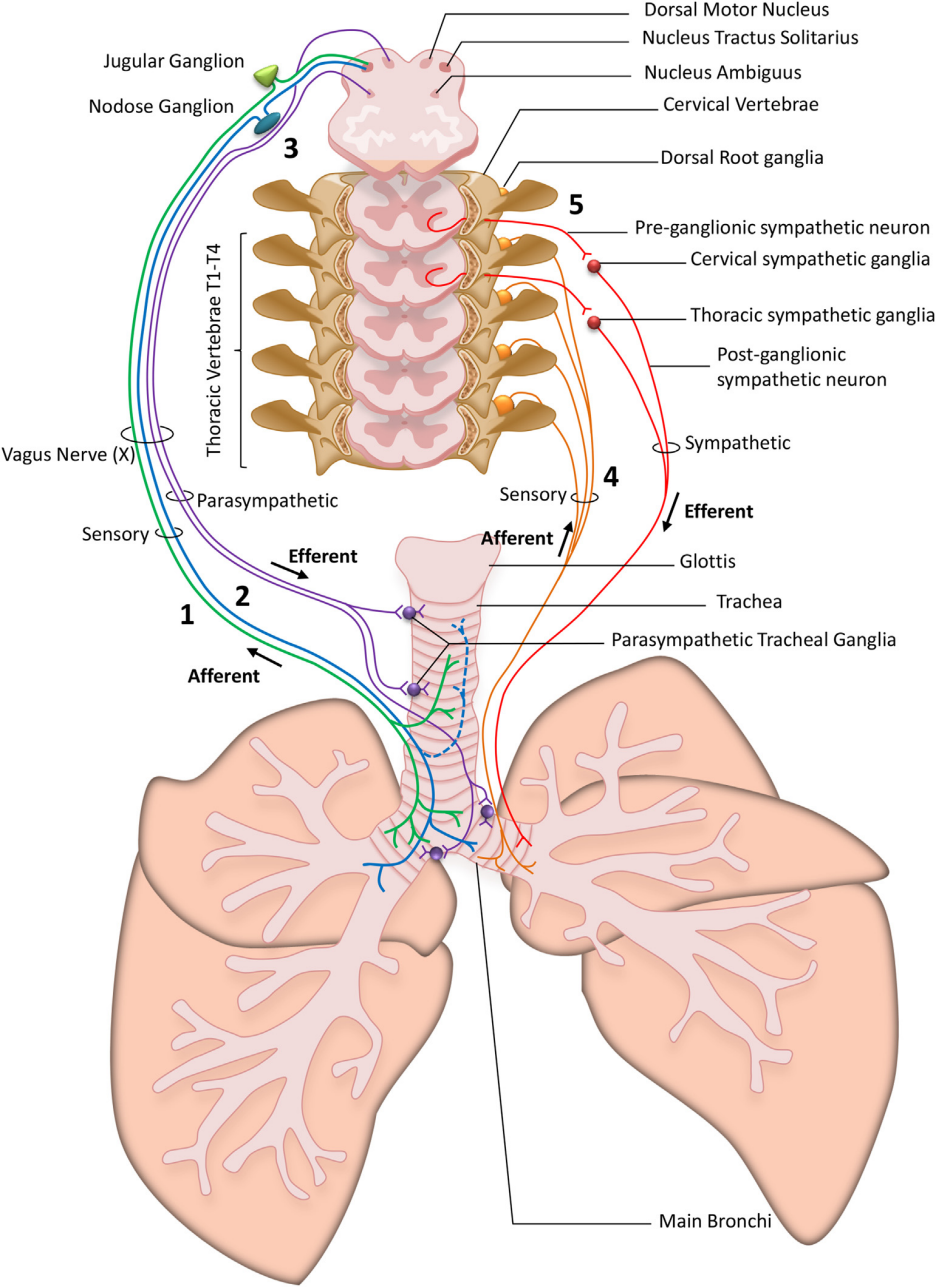
Schematic representation of airway innervation. The airways are innervated by a dual system of afferent sensory neurons and efferent motoneurons. The Vagus nerve (X) contains (1) the sensory neurons forming the C-fibres (green line) originating from the sub-cerebral jugular ganglion which innervate the upper and lower airways and project into the nucleus tractus solitarius and (2) neurons originating from the sub-cerebral nodose ganglia forming C-fibres which innervate the lower airway and bronchi (blue line) and ‘cough receptor’ mechano-sensitive Aδ fibres which innervate the upper airway (dashed blue line) and all also project to the nucleus tractus solitarius. (3) Two different parasympathetic pathways run alongside the sensory fibres nerves in the vagus nerve (X): Pre-ganglionic parasympathetic neurons originating from the nucleus ambiguous and the dorsal motor nucleus in the brainstem respectively innervate postganglionic cholinergic neurons located in tracheal parasympathetic ganglia. These parasympathetic ganglia regulate bronchial tone and mucus secretion. Nerves originating from the spinal cord also innervate the airways with (4) sensory neurons originating from the dorsal root ganglia in thoracic vertebrae T1 to T4 which innervate the lower airways and bronchi. These nerves are supposedly more pain orientated whereas their regulatory role in the airways is yet largely unknown. (5) Sympathetic pre-ganglionic neurons derived from the cervical and thoracic spinal cord respectively project to sympathetic neurones located in the cervical and thoracic ganglia. These sympathetic neurons innervate airways controlling smooth muscle tone and have been proposed to mediate bronchodilatation in some species (but not in human airways). (For interpretation of the references to colour in this figure legend, the reader is referred to the web version of this article.)

The cough reflex is mediated by sensory nerves which terminate in the upper and lower airways. These nerves differ in their origin (sensory ganglia), termination site (upper or lower airways), physiological characteristics (myelination, conduction speed, receptor expression) and sensitivity to stimuli. Fig. 1 summarises our current understanding of the different nerve fibres involved in cough. The vast majority of airway sensory nerve fibres originate from the vagal nodose and jugular ganglia which lie under the ear bone within the skull; with few coming from the thoracic dorsal root ganglia [17]. Nerve fibres terminate both in and under the airway epithelium. When stimulated by an irritant, ion channels on the nerve terminals open to allow cation influx. This leads to membrane depolarisation and subsequent opening of the voltage-gated sodium channels, thus generating an action potential. Action potentials are actively propagated along the nerve fibres, which are carried by the vagus nerve axon to the tract of the nucleus solitarius (NTS) where the fibres synapse. The cough pathway beyond the NTS is still unclear. It is believed that second order neurons then relay the message to a putative respiratory pattern generator which collates and organises the incoming information, and sends out an efferent ‘cough’ signal to co-ordinate respiratory muscle activity. Below is a brief discussion of the sensory nerves that innervate the respiratory tract, and their involvement in the cough reflex.

### 2.1 C-fibres

Airway C-fibres are activated by a wide range of chemical irritants. Stimuli that activate C-fibres range from food extracts (e.g. capsaicin, mustard oil, wasabi and ginger) to environmental irritants (e.g. cigarette smoke, air pollution and vehicle exhaust) and endogenous mediators (e.g. bradykinin, prostanoids, and products of oxidation) [18–24]. The chemosensitivity of C-fibres makes them attractive pharmacological targets for anti-tussive treatment, and they have therefore been a major focus in the search for novel cough therapies.

C-fibre afferents are non-myelinated and conduct with a slow velocity (<1 m/s). They originate mainly in the jugular vagal ganglia with some coming from the nodose ganglia, and mostly terminate within airway epithelium. It has been found that jugular C-fibres innervate both the upper (‘bronchial’) and lower (‘pulmonary’) airways; whereas C-fibres originating from the nodose and dorsal root ganglia terminate predominantly in the lower airways [1,17,25–28]. In addition to their physiological characteristics, C-fibres can be differentiated by their activation properties. For example, both jugular and nodose airway C-fibres express Transient Receptor Potential Vanilloid 1 (TRPV1) and respond to the irritant capsaicin [17,27–30]. Whereas, only nodose C-fibres express purinergic and 5-HT receptors and respond to adenosine and 5-HT [27,28,31,32]. The TRP receptors are a large family of cation channels, of which TRPV1 and TRPA1 (Ankyrin 1) have been identified as pro-tussive mediators [2,33–38]. The importance of these ion channels in modulating the cough reflex and their potential role in airway pathologies will be discussed in detail in Section 4.

Based on their presence in the upper airways, from which the cough reflex can readily be triggered, bronchial C-fibres are thought to initiate the cough reflex. By contrast, pulmonary C-fibres have been proposed to inhibit cough. For example, stimulation of pulmonary C-fibres in anaesthetised cats and dogs has been shown to inhibit cough induced by mechanical stimulation of the larynx or trachea [39,40]. Stimulation of pulmonary C-fibres triggers a chemoreflex that causes apnoea, rapid shallow breathing, bradycardia and hypotension. Apnoea is thought to suppress the respiratory rhythm generator and reduce expiratory efforts, during which time it is not possible to trigger cough [1,26,39,40]. An inhibitory sensory drive that regulates cough is an interesting concept, and liberation of an inherent inhibition could help to explain why people who suffer from airway disease exhibit an enhanced tussive reflex. The interaction between activation of stimulatory and inhibitory fibres could also help to explain the different patterns of cough elicited by different irritant stimuli [1]. However, there is still no sound evidence for such an inhibitory drive within the airways; and an augmented response to hazardous or innocuous stimuli could similarly explain the enhanced cough reflex associated with respiratory pathologies. If indeed pulmonary C-fibres do inhibit the cough reflex, then selective pharmacological activation of these receptors could be a useful therapeutic tool [1].

### 2.2 Aδ cough receptors

Irritant ‘cough receptors’ have been identified and differentiated from traditional low-threshold mechanically sensitive lung Aδ fibres [28,41,42]. Cough receptors originate in the nodose ganglia and largely terminate in the upper airways. By contrast to the heavily myelinated Aβ fibres (which are fast-conducting fibres at >14 m/s), the cough receptors possess a thin myelin sheath, and are therefore moderately fast-conducting (4–6 m/s) neurons. They are only modestly sensitive to mechanical stimulation and respond to acid challenge, but are not normally activated by capsaicin or bradykinin [42,43]. However, several studies suggest that Aδ fibres which terminate in the tracheal mucosa can be induced to transiently express neurokinins and TRPV1, allowing them to respond to capsaicin stimulation in models of airway disease [44–47]. These studies highlight the cough receptors as a potentially important target for hypersensitivity associated with respiratory pathologies.

### 2.3 Rapidly adapting receptors (RARs) and slowly adapting receptors (SARs)

RARs and SARs are mechano-sensitive afferent fibres that conduct in the ‘A’ range (>14 m/s), and were named for their distinct adaptive properties to lung inflation i.e. during inspiration RARs will typically show a short burst of action potentials that rapidly adapt within 1–2 s; whereas, SARs will continue to fire action potentials until lung volume returns to normal [26,43]. Both of these afferents terminate in the intrapulmonary airways, and are not thought to be directly involved in the cough reflex. Early work suggesting a role for RARs came mainly from studies on anaesthetised animals [1,39]; however it has subsequently been shown that substances which stimulate RARs under anaesthesia (e.g. substance P and histamine) are ineffective at evoking cough [28,42]. Though unlikely to be directly involved, this does not preclude the possibility that RARs and SARs could play an indirect role in modulating the cough reflex.

## 3 Methods used to investigate the cough reflex

A number of *in vitro* and *in vivo* models have been developed to study the tussive reflex. This section will briefly examine some of these models, their advantages and inherent limitations.

### 3.1 Calcium imaging of sensory ganglia

This preparation measures activation of primary neuronal cell bodies by assessing changes in intracellular calcium levels via fluorescent imaging [48–50]. Initially, the primary neuronal cells are isolated and adhered to an imaging plate. The cells are then loaded with a membrane-permeable dye which fluoresces under a specific wavelength of light when bound to calcium. As discussed in Section 2, the airway afferent fibres have their neuronal cell bodies located in the vagal and dorsal root ganglia. Stimulation of these neurons by an irritant triggers the opening of ion channels on the cell membrane, leading to depolarisation. If this depolarisation reaches a critical threshold, voltage-gated sodium channels also open, which in turn leads to opening of the voltage-gated calcium channels. Furthermore, opening of certain ion channels can lead to release of intracellular calcium stores from the endoplasmic reticulum. Under normal resting conditions the intracellular concentration of calcium is very low, and thus very little fluorescence is measured. Upon stimulation calcium will either enter the cell via activated ion channels, or be released from intracellular stores, and bind to the dye leading to an increase in fluorescence.

One of the major advantages of this model lies in the ability to measure from primary cells that project nerve fibres specifically to the airway. This is possible by instilling the fluorescent dye DiI (DiIC18(3),1,1′-dioctadecyl-3,3,3′,3′-tetramethylindocarbocyanine perchlorate) into the lungs, which is then retrogradely transported along the nerve fibre toward the neuronal cell body [47,51]. Neurons that have taken up this dye can then be identified via fluorescence before beginning experimentation. Further advantages include the ability to image from multiple species, including genetically modified mice; and the ease with which pharmacological studies can be performed. Conversely, this model is limited by the fact that recordings are made from the neuronal cell bodies, not the nerve terminals. It is therefore uncertain whether the responses being recorded represent what would happen at the nerve ending due to possible differences in protein expression, intracellular signalling or the presence/absence of secondary signalling molecules. It is also not known whether phenotypical changes occur during the cell isolation process.

### 3.2 Isolated vagus nerve depolarisation assay

This method involves removing the axon of the vagus nerve which runs caudal to the nodose ganglion. The nerve axon is then placed in a recording chamber, where the two ends of the nerve are electrochemically isolated, and recording electrodes are placed at either end – one recording the resting potential, and the other recording nerve activity upon stimulation. Nerve activation is measured as the compound change in membrane depolarisation (in millivolts), and indicates the sum of the activity of all fibres carried by the vagus nerve.

The isolated vagus preparation is a relatively high-throughput *in vitro* method to study native receptors and ion channels that has been shown to parallel cough responses seen *in vivo* and in the clinic in that, in general, agents that cause cough also depolarise the vagus [37,50,52]. It is also possible to produce translational data by utilising human vagal tissue, giving an indication of the similarities and differences that occur between humans and animal models. Furthermore, it is possible to use tissue from genetically modified animals, and assess pharmacological modulation of agonist responses. The main limitation of this model lies in the fact that you are recording from the trunk of the vagus nerve, which carries all types of afferent nerve fibres (RAR, SAR, cough receptor and C-fibre) as well as parasympathetic and potentially inhibitory nerves, and nerves innervating other visceral organs such as the heart and gastrointestinal tract. Moreover, as with the calcium imaging method, the effect observed on the isolated vagus trunk does not necessarily represent what is happening at the nerve terminals within the airway. It is also important to note that depolarisation of the vagus axon does not necessarily relate to action potential firing, as a certain threshold of depolarisation needs to be reached in order to generate the all-or-nothing action potential response.

### 3.3 Single fibre recording from airway afferents

A variety of *in vitro* and *in vivo* techniques exist for the single fibre model. In all of these models, the aim is to detect action potential firing from a single nerve fibre, whereby you can measure the frequency, duration and pattern of firing [43–45,53,54]. Depending on the model, it is possible to determine the type of fibre (C-fibre, cough receptor, RAR or SAR), the ganglion in which the fibre originates (nodose or jugular), and its termination site in the airways (bronchial or pulmonary). One of the major advantages of this technique is that you are recording actual firing of the nerve fibre, which gives a better indication of whether a stimulus is indeed causing information to be sent to the CNS. However, this technique is not as robust as other *in vitro* techniques, it is slow through-put (especially if looking for a particular type of nerve fibre), and it is difficult to conduct classical pharmacological characterisation.

### 3.4 In vivo cough models

The dog, cat, rabbit and guinea-pig have been used for investigating the cough reflex *in vivo*. Of these, the guinea-pig is currently the most common model due to its size (therefore requiring smaller amounts of compound), and the ability to easily perform experiments on conscious animals. Smaller rodents such as the rat and mouse do not exhibit a cough reflex that resembles human cough, and as such have rarely been used to conduct *in vivo* research [56].

A large number of experiments have been conducted using anaesthetised animals. Importantly, our current understanding of the nerves that regulate the cough reflex comes primarily from work using anaesthetised preparations where afferent nerve activity can be measured at the same time as tussive stimuli are applied. The use of anaesthetised animal models caused some debate during early investigation into the tussive reflex, with regard to whether C-fibres truly play a role in mediating cough. This was prompted by the observation that the cough reflex could be elicited by mechanical or acidic stimuli both in conscious or anaesthetised animals; whereas chemical stimuli such as capsaicin and bradykinin that caused coughing in conscious animals failed to induce cough under anaesthesia [40,42]. Moreover, there was evidence to suggest that pulmonary C-fibre stimulation actually inhibited the cough response [1,26,39,40]. It is now widely accepted that C-fibres are important in regulating cough, and that anaesthesia somehow suppresses neuronal conductance. This is an important limiting factor in the anaesthetised preparation, as C-fibres currently provide the most promising pharmacological target for novel anti-tussive therapies.

More recently, *in vivo* cough experiments have been conducted using conscious guinea pigs, and a great deal of information has been gathered with regards to the pharmacological modulation of cough in this way. Briefly, conscious guinea pigs are placed unrestrained in to a Perspex chamber prior to exposure to aerosols of tussive stimuli. A tussive effort is determined by the change in airflow in the box (measured and recorded via software), and by the distinctive sound (recorded via microphone) and posture adopted by the animal [36–38,48,50,52]. In this way, the number of coughs generated by a tussive stimulus can be counted, and augmentation or inhibition of the response can be determined with pharmacological intervention, or during induction of a pathological state (see disease models section). The guinea pig tussive reflex has also been shown to display similarities to human cough [4,33,37].

### 3.5 Disease models

The vast majority of research on cough has been conducted on naïve animals. This research is inherently important, as we need to understand how the cough reflex works in the healthy state in order to then determine what alterations occur that lead to excessive cough in pathological states. Advances have recently been made in the area of disease research which are beginning to clarify the phenotypic, genotypic and physiological changes that may lead to chronic cough. These studies have been conducted in animal models of smoke inhalation, viral infection, and allergy. On the whole these models demonstrate an increase in cough reflex sensitivity to tussive agents like capsaicin rather than an increase in spontaneous coughs as guinea pigs do not appear to cough unless challenged with a tussive agent.

Sub-chronic (up to two weeks) [55–58] and chronic models (90 days) [59] of cigarette smoke exposure have been successfully used to induce enhanced cough reflexes upon subsequent stimulation with tussive agonists (mainly capsaicin). In these studies, animals were placed unrestrained in to a chamber and exposed to smoke or room air for a defined number of cigarettes or period of time. These studies successfully induced hypersensitivity to tussive stimuli in guinea pigs and as such may be useful as a model of enhanced cough in the context of smoking related diseases. However, the sub chronic smoke exposure is unlikely to have induced some of the more permanent and severe physiological changes in the lungs that are associated with diseases such as emphysema and COPD, for example inflammation, destruction of lung parenchyma and fibrosis. The authors of this paper are aware of only one study looking at chronic exposure to cigarette smoke, probably due to the prohibitive costs involved with long-term research. Histological examination of the tracheal epithelium in these animals revealed airway inflammation, and an increase in the number of alveolar macrophages and eosinophils which was associated with a similar increase in coughs to capsaicin challenge as had been seen in the sub chronic model [59]. The results of these studies could therefore be consistent with smoking related pathologies in the clinic.

Upper respiratory tract viral infection is one of the most common causes of cough hypersensitivity. Even in healthy individuals, cough due to viral infection may last for several days or weeks after other symptoms have ceased and the infection has been cleared. This phenomenon is termed post-viral cough and suggests a transient sensitisation of the respiratory tract that may or may not possess immunomodulatory benefits. It is also hypothesised that chronic idiopathic cough may stem from long-term irreversible potentiation of the cough reflex following a respiratory tract infection which has long since been cleared. This could account for the fact that chronic idiopathic cough patients do not present with any underlying condition. Moreover, viral models are important to study the possible pathologies associated with virally-induced asthma and COPD exacerbations, which are associated with a high rate of hospital admissions in patients [60]. Animal models developed to investigate viral infections involve intranasal inoculation with virus-containing solution or vehicle, normally under anaesthesia. The animals are then left to recover while viral infection is allowed to develop over several days. Two different guinea pig models of viral infection (sendai virus and parainfluenza 3 virus) have been observed to cause phenotypical changes in airway afferent nerve fibres, and enhanced cough responses to capsaicin [44,61].

The guinea pig model of asthma is one of the oldest used to investigate allergic airway responses. The standard model of allergy is to sensitise and challenge animals with ovalbumin, which generates eosinophilia and increased airway responsiveness, similar to an asthmatic phenotype [62]. It has also been shown that there are similarities between guinea pig and human airways in the allergic response to methacholine, histamine and allergen challenge following sensitisation [62]. However, differences also exist, including the response to leukotriene challenge, and the fact that guinea pigs produce mainly immunoglobulin G1 antibodies, in contrast to immunoglobulin E1 in humans. Furthermore, there are multiple methodologies used with respect to sensitisation, route and duration of challenge, which may affect the endpoint measurements. Despite these differences in approach, the guinea pig model of allergy has been shown to induce phenotypical changes in the nerve fibres that mediate cough [45–47], which may lead to cough hypersensitivity.

### 3.6 Clinical trials

The lack of success in clinical trials investigating promising anti-tussive targets which were identified using some of the above animal models has led to the perception that these models are not predictive of human cough. However, the vast majority of clinical trials investigating the cough reflex are not conducted under ideal conditions. Some of the main underlying problems with clinical trials are that cough is rarely the primary endpoint of the study, that cough is not quantitatively or objectively measured, subject numbers are too small to provide appropriate statistical power, and that many trials do not include the appropriate controls. Furthermore, there are more restrictions placed on clinical trials in terms of dose, route of administration, and CNS penetration of potential anti-tussive therapies. It is also worth considering that antagonist studies in animal models are usually carried out on healthy animals; whereas some clinical trials attempt to treat cough in patients suffering a variety of respiratory pathologies. This is difficult not only in terms of diagnosis of the respiratory pathology and grouping of patients (especially in the case of co-morbidities), but we need to consider that any potential beneficial effect in one group of subjects may be masked by the lack of effect in another group.

Classically, clinical trials investigating potential anti-tussives have been conducted either qualitatively via a series of questionnaires based on an individual's perception of their cough using visual scales; or quantitatively using cough sensitivity to a particular tussive ligand (usually capsaicin or citric acid) where the outcome measure is the concentration of pro-tussive stimulus required to cause either two (C2) or five (C5) coughs. Using subjective measures of cough introduces large amounts of bias, as the outcome relies on the perception of individuals, which can be affected by a number of environmental factors and are inherently unreliable. Moreover, determining the number of coughs induced by inhaled substances such as citric acid or capsaicin may not be applicable to real-world situations. This is especially true for chronic coughers where excessive cough may be caused by the release of endogenous mediators. However, a non-invasive objective cough monitor was recently developed which measures cough sounds that are recorded and subsequently quantified. This objective 24 h monitoring of cough could provide more constructive and less biased information on cough severity and the effectiveness of anti-tussive treatments in a real-world setting, which may be more useful in identifying new therapies [63].

## 4 Transient receptor potential ion channels and cough: history and recent developments

**Fig. 2:**
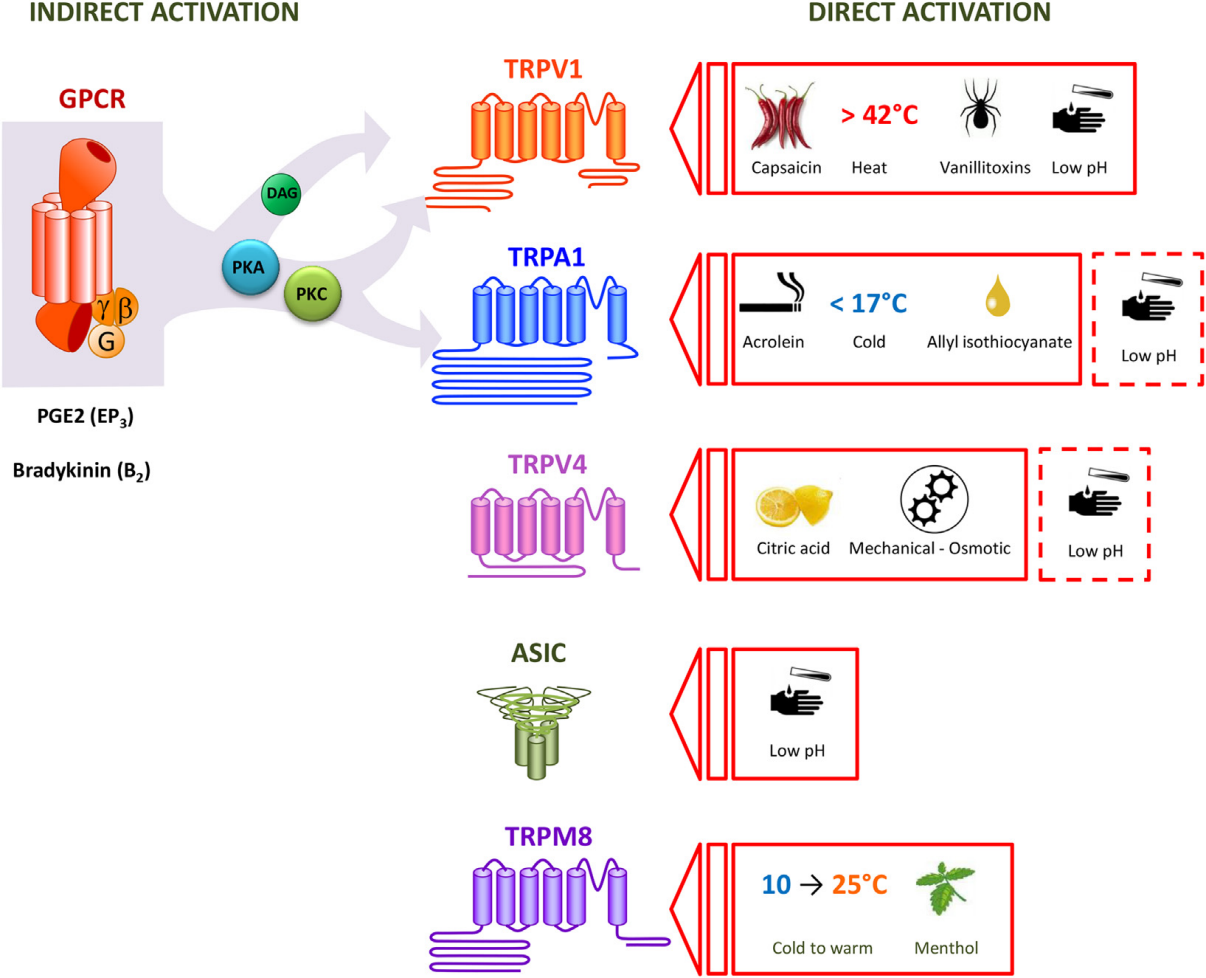
Schematic of the principal ion channels acting as chemosensors in airway sensory neurons: Transient Receptor Potential Vanilloid 1 (TRPV1); Transient Receptor Potential Ankyrin 1 (TRPA1); Transient receptor potential vanilloid 4 (TRPV4); Acid sensing ion channels (ASIC); Transient receptor potential melastatin 8 (TRPM8). Substances known as directly activators of the channels are displayed on the right. Known indirect activators of the channels which bind G protein coupled receptors (GPCR) leading to activation of TRP channels via production of diacylglycerol (DAG) and activation of Protein Kinase C (PKC) by phospholipase C (PLC) are indicated on the left of the figure. The dashed lines indicate the fact that the TRPA1 and TRPV4 ion channels are also purported to sense changes in pH.

The original *Trp* channel was isolated from the drosophila fly, and named for its transient rather than sustained response to bright light [64]. Since then 28 mammalian TRP channels have been discovered, which are activated by intracellular and extracellular messengers, chemical compounds, mechanical stimuli, temperature changes and osmotic stress [65]. Many of the TRP channels have been linked to sensory perception, and are associated with the pathogenesis of a range of diseases including respiratory pathologies such as COPD, asthma, cancer and cystic fibrosis [19,66–68]. As such, a great deal of research has recently focused on the TRPs as pharmacological targets (Fig. 2).

Several TRPs are expressed in the airways, and modulate inflammation, airway smooth muscle tone, and activation of sensory afferents [69,70]. Specifically, the TRPA1 and TRPV1 ion channels have an established role in cough. TRPV1 (previously Vanilloid Receptor 1, or VR1) was cloned and characterised in 1997 [19], and was the first TRP to be identified as a mediator of the tussive reflex [34]. Like other TRP channels, TRPV1 is a polymodal sensor that is activated by a diverse range of agonists. These include direct agonists such as capsaicin [19], heat [19], low pH [71], and anandamide [72–74]; and indirect agonists which bind to G protein-coupled receptors (GPCRs) on the cell membrane and initiate intracellular signalling cascades that activate TRP channels. Indirect agonists include the inflammatory mediators bradykinin [50,75,76], and prostanoids such as Prostaglandin E_2_ (PGE_2_) [50,77]. Two well-known TRPV1 agonists, capsaicin and citric acid, are powerful tussive agents which reliably produce cough in both animals and humans, and these stimulants are regularly used in clinical assessment of cough sensitivity [2,4,9,33–36,78]. By contrast, TRPA1 (formerly ANKTM1) was first isolated from human fibroblasts in 1999 [79] and later found to be expressed in a subset of TRPV1-expressing small diameter nociceptive neurons [80,81]. In 2009 it was discovered that activation of the TRPA1 ion channel also causes cough in humans and animals [37,38]. This finding could be of particular significance because many of the chemicals that are known to activate TRPA1 are noxious respiratory irritants, for example constituents of pollutants such as diesel exhaust, wood and cigarette smoke, burning vegetation, and isocyanates which have been linked to the late asthmatic response [20,82,83]. Similar to TRPV1, TRPA1 is activated by endogenous substances released during inflammation (bradykinin and PGE_2_ via GPCR activation) and oxidation (4-hydroxy-nonenal, 4-oxynonenal) [20,22,23,50,80,84–86].

In response to tissue injury and inflammation, the body releases endogenous compounds that mediate a wide range of effects. This response is thought to form the basis for development of hypersensitisation in disease states. For example, release of inflammatory mediators such as PGE_2_ and bradykinin in the airways may lead to sensitisation of the cough response to tussive stimuli [87,88]. In agreement with this hypothesis, PGE_2_ and bradykinin have been shown to sensitise human and animal cough responses to TRPV1 irritants and enhance sensory nerve fibre responses *in vitro* [87–91]. Notably, PGE_2_ and bradykinin also cause cough in conscious animals and humans when inhaled as an aerosol [52,86,92,93]. This suggests that these irritants are not only sensitising the cough reflex, but are capable of activating it. Therefore, it is possible that enhanced release of endogenous ligands during disease states may lead to levels high enough within the lungs to activate the tussive reflex without the need for an outside stimulus [50]. This is supported by data from patients taking angiotensin-converting enzyme inhibitors, who experience excessive cough apparently due to reduced breakdown (and therefore accumulation) of bradykinin [88].

As indicated above, PGE_2_ and bradykinin bind to GPCRs. GPCRs are a family of receptors that initiate a diverse range of intracellular signalling cascades depending on what G-protein the receptor is coupled to. PGE_2_ has been shown to cause airway nerve activation and cough via the EP_3_ receptor [52]; and bradykinin via the B_2_ receptor in guinea pigs and humans [50]. However, some species differences do occur, for example in a murine model of isolated sensory nerve depolarisation both the B_1_ and B_2_ receptors were shown to be important for bradykinin-induced stimulation [50]. Irrespective of the GPCR or signalling cascade stimulated, an ion channel would subsequently need to be activated in order to generate nerve firing. Indeed, it was recently shown that the tussive response to both PGE_2_ and bradykinin are partially mediated by the TRPA1 and TRPV1 ion channels [50], leading the authors to propose that TRPA1 and TRPV1 could be thought of as ‘common effectors’ of the tussive response to endogenous mediators. Interestingly, TRPA1 or TRPV1 antagonism produced a much greater inhibition of the PGE_2_ or bradykinin-induced cough reflex *in vivo* than would have been expected from the approximately 50% inhibition observed on isolated sensory nerves *in vitro* [50]. This could be due to the nature of the peripheral nervous system, whereby inhibition of membrane depolarisation by 50% may lead to a much greater inhibition of action potential discharge (due to their all-or-nothing characteristic) and therefore a substantially reduced ‘cough’ signal being sent to the CNS. Alternately, there could be co-operation between the TRPA1 and TRPV1 ion channels *in vivo* that is not being observed in the *in vitro* preparations. In any case, the use of a single inhibitor may allow for reduced tussive responses while still preserving the overall ability to respond to tussive stimuli; in contrast to combined TRPA1/TRPV1 inhibitors which would wipe out the cough response to these endogenous tussive agonists.

A decrease in lung pH has also been observed in respiratory pathologies associated with an enhanced cough reflex [94,95]. The balance of pH within the body is normally maintained within a narrow range in healthy individuals, and it is therefore not surprising that acidic stimuli activate C-fibre and Aδ nerve fibres leading to activation of the cough reflex [4,28,34,42,76]. Low pH-induced cough is known to be partially mediated via the TRPV1 ion channel [34,96,97]. However, it has yet to be definitively established what other ion channel(s) also play a role. The most likely candidates are members of the Acid Sensing Ion Channel (ASIC) family, and there is some *in vitro* evidence supporting this claim [96,97]. However, the TRPA1 and TRPV4 ion channels are also purported to sense changes in pH [98,99]. Research investigating the tussive effects of low pH is complicated by the likelihood that different levels of pH may be mediated by different ion channels (akin to temperature where different TRP channels are activated by certain ranges of heat or cold). This is compounded by the inability to determine what level of pH is actually reaching the nerve endings within the airways, due to the ability of the respiratory tract to buffer inhaled substances. Moreover, the tools currently available which modulate the ASIC ion channels are inadequate.

Further to the above discussion, it is still unclear whether there is cooperation between TRPV1 and TRPA1 channels. Both are activated by tussive agents and seem to be commonly activated downstream of GPCR coupling, so it could be possible that they act in concert to elicit functional responses. It has also been suggested that TRPA1 channels can be activated by an overflow of calcium in the locale of other activated channels or via release of intracellular stores from the endoplasmic reticulum, without TRPA1 ever being modified by a reactive ligand [100,101]. There is evidence for this type of coupling with bradykinin signalling in trigeminal neurons [20]. However, whether TRP channel co-operation exists in generating a cough reflex has yet to be determined.

## 5 Other TRPs that could be involved in the cough reflex

There is an expanding body of literature investigating the role of TRPA1 and TRPV1 in modulating airway sensory afferents and the tussive reflex [37,38,43,47,50,69,102]. By contrast, the role of other TRP channels in the airways is relatively unexplored. TRPM8 (Melastatin 8) is a temperature sensor which is activated in the ranges of innocuous (26–15 °C) to noxious cold (<15 °C), and by cooling compounds such as menthol and icilin [103,104]. TRPM8 has been suggested to be the ion channel responsible for cough and airway constriction associated with inhaling cold air, however this has yet to be substantiated [104,105]. There is also conflicting data on the anti-tussive effects of TRPM8, with some studies suggesting that menthol inhibits the cough reflex [107–109]. Though its efficacy as an anti-tussive is uncertain, menthol is widely used in over-the-counter cough therapies [106–109]. The conflicting data on TRPM8 is confounded by the lack of selective tools, as both menthol and icilin are known to activate TRPA1 at higher concentrations. There have recently been a number of novel TRPM8 inhibitors developed, but these compounds are yet to be thoroughly validated [106].

The TRPV4 ion channel has also recently become of interest in the respiratory field, as there are polymorphisms of this channel associated with both COPD and cystic fibrosis [66,68]. TRPV4 is expressed in airway tissues, including airway smooth muscle, the alveolar wall, lung tissue, lung vessels and inflammatory cells [110–113]. Moreover, TRPV4 is expressed in DRG neurons [114], suggesting that it may also be expressed on the sensory nerve endings of the airway. TRPV4 was originally characterised as a sensor of osmotic and mechanical stimuli [115,116]. Similar to TRPA1 and TRPV1, TRPV4 is also known to be sensitised by PAR2 signalling, resulting in increased sensitivity to painful mechanical stimuli [114]. This data suggests that dysfunction of TRPV4 may be involved in the pathogenesis of airway disease, and could play a role in sensitisation of afferent nerves, which could hold implications for cough pathologies.

## 6 Disease relevance and problems in the clinic

The majority of pre-clinical cough research has thus far focused on sensory afferents, receptors and ion channels that are mediating the cough reflex under healthy conditions. However, it is chronic cough associated with respiratory disease that is driving the call for novel and effective anti-tussive therapies. Indeed, now that researchers have begun to elucidate the mechanisms that drive the cough reflex in the healthy state, emphasis has begun to shift towards trying to understand what is changing in disease states to lead to hypersensitive pathologies. As you would expect, the TRP ion channels are the main focus of this research.

In pre-clinical models, both chronic and sub-chronic exposure to cigarette smoke have been observed to cause an increase in the number of coughs caused by stimulation with capsaicin or citric acid in guinea pigs *in vivo* [56–59], which corresponds with enhanced sensory nerve reactivity to these TRPV1 stimuli *in vitro* [58]. Moreover, animal models of viral infection have been shown to lead to changes in airway nerves and an enhanced cough reflex. For example, infection of guinea pigs with sendai virus was shown to induce transient neurokinin expression in the nodose neurons of large cell body diameter (a feature of non-nociceptive afferents) and which project nerve fibres to the trachea. Expression of neurokinins is normally a hallmark of nociceptive C-fibre afferents that also express the TRPV1 ion channel. This is an important discovery, suggesting that viral infection induces a phenotypic change in the vagal afferent innervation of the airway, which could lead to an enhanced cough reflex [44]. Unfortunately cough was not directly investigated in this study. However, it has recently been shown by another group that infection with parainfluenza 3 virus does indeed lead to cough hypersensitivity in guinea pigs subsequently exposed to aerosolised capsaicin [61]. Interestingly, Ye and colleagues observed significantly enhanced cough sensitivity in the viral treated group compared to control through to post infection day 42, even though the viral infection had all-but cleared by this time. This suggests that the animals developed “post-viral cough” in their disease model [61]. Similar to viral infection, animal models of allergy have shown a phenotypic switch in neurons of large cell body diameter, which begin to express neurokinins [45]. The same group also recently observed that Aδ-type fibres can be induced to express TRPV1 ion channels during allergic inflammation [47]. Together the above data paints the picture of a plastic and flexible peripheral nervous system, which is capable of adapting to its environment and expanding the range of stimuli capable of activating certain nerve fibres. This is likely to be important in the development of chronic pathologies, for which TRPV1 and possibly other ion channels of the same family appear to play a vital role.

In the clinic it has been established that chronic coughers of varying aetiologies exhibit hypersensitivity to capsaicin challenge in comparison to healthy controls. Some of the pathologies linked to capsaicin hypersensitivity include asthma, COPD, rhinitis, interstitial lung disease and upper respiratory tract infections [4,9,35,78,117–121]. Moreover, expression of TRPV1 in the lung is increased in patients who suffer from chronic cough [122]. These findings implicate TRPV1 in the pathogenesis of chronic cough associated with respiratory diseases and have highlighted TRPV1 as a potential novel therapeutic target. However, in clinical trials investigating TRPV1 antagonists as potential analgesics, it was discovered that inhibition of this ion channel with certain TRPV1 antagonists causes hyperthermia [123,124]. This is a potentially confounding effect in the development of TRPV1 therapies, and there is now a dedicated search for efficacious TRPV1 inhibitors that do not affect body temperature [124–126].

TRPA1 has been implicated in pain hypersensitivity [127,128] and the late asthmatic response [102], though data on an equivalent role for TRPA1 in excessive cough is currently lacking probably due to the lack of commercially available tools. Clinical trials investigating TRPA1 as a target for cough are yet to be attempted due to the poor selectivity and potency of first generation antagonists [129]. Similarly, selective antibodies for TRPA1 are not currently available, and so studies have not yet been conducted to show if TRPA1 expression changes under pathological conditions associated with enhanced cough in man. These studies are important, as evidence would suggest that TRPA1 will be just as important as TRPV1 in cough associated with disease, if not more-so due to the types of irritant that bind to and activate TRPA1. With the recent development of better pharmacological tools, studies investigating the role of TRPA1 in pre-clinical models of cough pathologies will be a priority, and hopefully clinical trials will be forthcoming in the near future.

## 7 Summary

In the last two decades, scientists investigating cough have made come a long way in understanding the mechanisms driving this reflex, and how it is being modulated by both endogenous and exogenous compounds. Most recently, findings have linked TRPA1 and TRPV1 as possible ‘common effectors’ of the tussive response downstream of GPCR coupling by inflammatory mediators such as PGE_2_ and bradykinin. Moreover, a variety of TRP channels as well as ASICs are proposed to be associated with the sensation of acidic stimuli (e.g. TRPA1, TRPV1 and TRPV4). Pre-clinical and clinical evidence continues to demonstrate a role for these ion channels as potentially important targets for the treatment of cough associated with respiratory pathologies.
